# Immune Profiles in Multisystem Inflammatory Syndrome in Children with Cardiovascular Abnormalities

**DOI:** 10.3390/v15112162

**Published:** 2023-10-27

**Authors:** Nathella Pavan Kumar, Aishwarya Venkataraman, Arul Nancy, Nandhini Selvaraj, Kadar Moideen, Shaik Fayaz Ahamed, Rachel Marriam Renji, Kandasamy Sasidaran, Sandip Kumar, Muthiah Periyakuppan, Thankgavelu Sangaralingam, Poovazhagi Varadarajan, Elilarasi Chelladurai, Subash Babu

**Affiliations:** 1 ICMR—National Institute for Research in Tuberculosis, Chennai 600031, India; venkataraman.a@icmr.gov.in (A.V.); ahamedsfayaz@gmail.com (S.F.A.); 2 National Institutes of Health-National Institute for Research in Tuberculosis-International Center for Excellence in Research, Chennai 600031, India; arul.p@icerindia.org (A.N.); nandhu30797@gmail.com (N.S.); kadarbinabbas@gmail.com (K.M.); rachelmr1610@gmail.com (R.M.R.); sbabu@icerindia.org (S.B.); 3 Dr. Mehta’s Children’s Hospital, Chennai 600031, India; sasidarpgi@gmail.com (K.S.); prmuthiah@gmail.com (M.P.); thangavelu.dr@gmail.com (T.S.); 4 Institute of Child Health and Hospital for Children, Chennai 600008, India; poomuthu@gmail.com (P.V.); elil.raghu@gmail.com (E.C.); 5 Laboratory of Parasitic Diseases, National Institute of Allergy and Infectious Diseases, National Institutes of Health, Bethesda, MD 20892, USA

**Keywords:** MIS-C, cytokines, chemokines, cardiac manifestation, SARS-CoV-2

## Abstract

Background: Multisystem inflammatory syndrome in children (MIS-C), a sequela of severe acute respiratory syndrome coronavirus-2 infection (SARS-CoV2), has been progressively reported worldwide, with cardiac involvement being a frequent presentation. Although the clinical and immunological characteristics of MIS-C with and without cardiac involvement have been described, the immunological differences between cardiac and non-cardiac MIS-C are not well understood. Methods: The levels of type 1, type 2, type 17, other proinflammatory cytokines and CC chemokines and CXC chemokines were measured using the Magpix multiplex cytokine assay system in MIS-C children with MIS-C cardiac (MIS-C (C) (*n* = 88)) and MIS-C non-cardiac (MIS-C (NC) (*n* = 64)) abnormalities. Results: MIS-C children with cardiac manifestations presented with significantly increased levels of cytokines such as IFN-γ, IL-2, TNFα, IL-5, IL-1α, IL-1β, IL-6, IL-10 and IL-12p70 and chemokines such as CCL2, CCL3, CCL11 and CXCL10 in comparison to MIS-C children without cardiac manifestations. Clustering analysis revealed that cytokines and chemokines could clearly distinguish MIS-C children with and without cardiac manifestations. In addition, these responses significantly diminished and normalized 9 months after treatment. Conclusions: This is one of the first studies characterizing and differentiating systemic inflammation in MIS-C with and without cardiac involvement from a low- and middle-income country (LMIC). Our study contributes to the existing body of evidence and advances our knowledge of the immunopathogenesis of MIS-C in children.

## 1. Introduction

Severe acute respiratory syndrome coronavirus 2 (SARS-CoV-2) infection is usually very mild or asymptomatic in children. However, multisystem inflammatory syndrome in children (MIS-C), a post inflammatory sequela of SARS-CoV-2 infection, presents with multiorgan dysfunction and significantly elevated markers of systemic inflammation, which may be potentially fatal [1,2,3,4]. The pathogenesis of MIS-C is still vague, but it is known that MIS-C presents with an underlying cytokine storm similar to Kawasaki disease (KD) and is likely due to an autoimmune etiology [5].

Approximately 18–87% of children with MIS-C have cardiovascular involvement [6]. The cardiovascular abnormalities in MIS-C include acute myocardial dysfunction, coronary artery dilatation, arrhythmia, ventricular dysfunction, conduction abnormalities, and rarely pericarditis and valvulitis [2,7]. Cardiovascular deterioration and shock can be severe and rapid in MIS-C [8]. However, a proportion of children with MIS-C present with no cardiovascular involvement. Nevertheless, when MIS-C is suspected, a thorough cardiac evaluation is paramount. There is a pressing need to elucidate the immunological aspects allied with MIS-C and decipher the similarities and differences in children with or without cardiovascular involvement, in order to understand the pathogenesis of MIS-C and guide treatment pathways. 

Our group has previously reported that MIS-C is a distinct and unique immunopathogenic pediatric illness related to SARS-CoV-2, presenting with elevated cytokines and chemokines which are different from acute COVID-19 and other hyperinflammatory conditions [9]. Therefore, we hypothesized that cardiac manifestations of MIS-C might also be associated with an altered cytokine and chemokine systemic milieu. Hence, the primary aim of this study was to elucidate the circulating levels of cytokines and chemokines in children presenting with MIS-C with or without cardiac involvement. Our findings reveal that MIS-C with cardiac involvement showed elevated cytokine and chemokine levels in comparison to non-cardiac MIS-C, implicating a potential association of these immune markers in cardiac pathology. 

## 2. Materials and Methods

### 2.1. Ethics Statement

Informed consent was obtained from parents/guardians of all children along with assent where appropriate. The Internal Ethics Committee (IEC) of the ICMR-National Institute for Research in Tuberculosis [Approval Code: 267/NIRT-IEC/2020 and 339/NIRT-IEC/2020, Approval Date: 23 October 2020 and 11 December 2020] approved this study.

### 2.2. Study Population and Procedures

We prospectively enrolled children admitted to the Institute of Child Health and Dr Mehta’s Children’s Hospital from 1 December 2020 to 30 March 2022 with MIS-C. Briefly, we included children of either sex between 1 and 18 years of age who were or whose parents were willing to provide informed consent/assent. Blood collection was performed prior to any immunomodulatory medication. Plasma was isolated and used to measure multiple immune parameters. Children with MIS-C were diagnosed according to the World Health Organization (WHO) case definition and all the enrolled MIS-C cases had no other microbial or viral inflammatory focus [10]. Children with comorbidities or other illnesses were excluded. SAR-CoV-2 serology was performed using the iFlash R SARS-CoV-2 IgG chemiluminescence antibody assay (CLIA) (YHLO Biotechnology Corporation, Shenzhen, China) according to the manufacturer’s instructions. An IgG antibody titer greater or equal to 10 AU/mL was considered positive.

Anemia was defined according to the WHO definition as hemoglobin <11 g/L up to 5 years, <11.5 g/L for 5–11 years and <12 g/L for 12–14 years [3]. Thrombocytopenia was defined as a platelet count <150 × 109/L, while thrombocytosis was defined as a platelet count >450 × 109/L, and lymphopenia was defined as a lymphocyte count <3.0 × 109/L [11]. Cardiac evaluation for all children was performed by a qualified pediatric cardiologist using a GE Vivid S6 ultrasound machine with a phased array cardiac transducer. Coronary artery abnormalities were categorized as normal (z score < 2), dilated (z score 2–2.5) or aneurysm (z score > 2.5), and aneurysms were sub-categorized as small, medium and giant based on their z scores (2.5–5, 5–10 and >10, respectively) [12]. An ejection fraction less than 55% was considered as indicating left ventricular dysfunction [13].

For analyses, a total of *n* = 152 children were included, and they were classified into two groups: MIS-C with (*n* = 88) cardiovascular involvement (MIS-C cardiac) and MIS-C without (*n* = 64) cardiac manifestations (MIS-C non-cardiac). Of the 152 children, a subset of 40 children were reviewed at 9 months after discharge. Blood was collected in EDTA tubes (BD Biosciences) as well as heparin tubes and processed within 4 h of collection at the National Institute for Research in Tuberculosis (NIRT), Chennai. Plasma was separated and stored and pooled analysis was performed for all samples together. Sampling in all children was conducted prior to receiving any immunomodulatory treatment. To avoid measurement bias and to increase the precision of the estimates for the accuracy of the assay, the study staff involved in immunological assays were blinded to any clinical data. All children were positive for SARS-CoV-2 IgG. They were diagnosed and treated according to the WHO guidelines and the Indian Academy of Pediatrics guidelines for MIS-C. 

### 2.3. Multiplex Assay

The levels of cytokines and chemokines were measured using the Magpix multiplex cytokine assay system (Bio-Rad, Hercules, CA, USA). The cytokines analyzed were IFN-γ, IL-2, TNF-α, IL-4, IL-5, IL-13, IL-17A, IL-1α, IL-1β, IL-6, IL-10, IL-12p70, IL-18 and granulocyte-macrophage colony-stimulating factor (GM-CSF), and the chemokines analyzed were CCL1, CCL2, CCL3, CCL4, CCL11, CXCL1, CXCL2, CXCL9, CXCL10 and CXCL11. These cytokines and chemokines were measured via ELISA using the kit from R&D Systems (Minneapolis, MN, USA).

### 2.4. Statistical Analysis

Geometric means (GM) were used for measurements of central tendency. Statistically significant differences between MIS-C with and without cardiovascular abnormalities were analyzed using the Kruskal–Wallis test with Dunn’s multiple comparisons. *p* ≤ 0.05 was considered statistically significant and all tests were two-sided. Wilcoxon signed rank test was used to compare levels of culture supernatant cytokine and chemokine concentrations before and after treatment. Analyses were performed using Graph-Pad PRISM Version 9.0 (GraphPad Software, Boston, CA, USA). R studio was used for plotting Spearman’s correlation, and the “Complex Heatmap” package in “RStudio 2023.06.1+524 was used to visualize and observe the difference in the expression of each of the cytokines and chemokines across the groups.

## 3. Results

### 3.1. Basic Demographics of MIS-C Children

**Figure 1:**
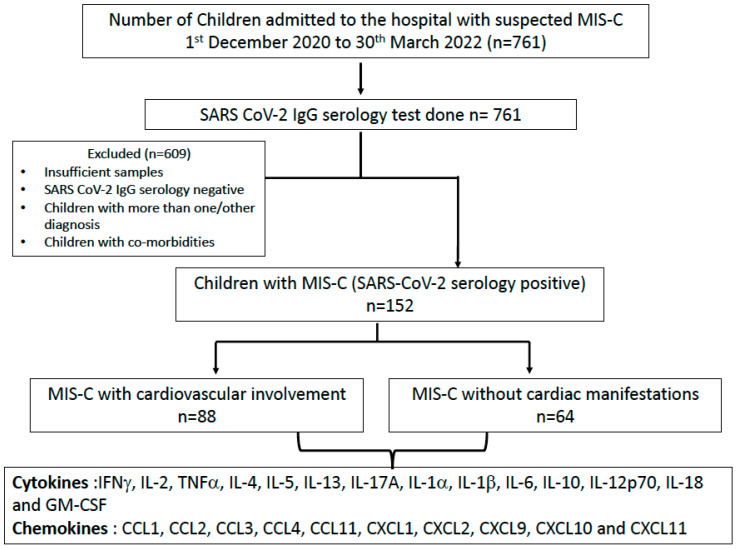
Outline of participant categorization. In the study cohort (*n* = 152), plasma samples were collected from children who were MIS-C with (*n* = 88) cardiovascular involvement (MIS-C cardiac) and MIS-C without (*n* = 64) cardiac manifestations (MIS-C non-cardiac).

The recruitment algorithm for the children for the study cohort is shown in Figure 1. A total of 761 children were admitted with suspected MIS-C, of which 152 children were included in this study. All 152 children, with a median age of 6 years (IQR 8, 1.25 years), 51% (78/152) males, were positive for SARS-CoV2 IgG. Excluded children were children with more than one diagnosis or other diagnoses, those with an insufficient sample for immune assays and/or those who were negative for SARS-CoV2 IgG. Of the 152 children, 58% (88/152) presented with one or more cardiovascular abnormalities (MIS-C cardiac) and 42% (64/152) had no cardiovascular involvement (MIS-C non-cardiac). Cardiovascular involvement included shock (80%, 70/88), myocardial dysfunction (50%, 44/88), coronary dilatation (25%, 22/88) and pericarditis (5%, 4/88). The mortality rate was approximately 2% (3/152). Among the 88 children with cardiovascular involvement (MIS-C cardiac), 80% (70/88) had other systems’ involvement, predominantly the gastrointestinal and/or mucocutaneous system. The basic demographics and laboratory investigations of these children are detailed in Table 1. Children in both the groups received treatment with immunomodulatory drugs; either steroids or IVIG or both. Nearly 98% (86/88) of MISC-cardiac patients required intensive care support, in comparison to 31% (20/64) of MIS-C non-cardiac patients.

### 3.2. MIS-C Children with Cardiovascular Abnormalities Have Altered Biochemical Profiles

To determine the changes in the hematological and biochemical profiles between MIS-C cardiac and MIS-C non-cardiac children, we assessed the hematological and biochemical indices in whole blood or serum/plasma. As shown in Table 1, lymphocyte counts (*p* < 0.0001), platelet counts (*p* < 0.0001), ProBnP (*p* < 0.005) and troponin levels (*p* < 0.005) were significantly elevated in the cardiac MIS-C patients in comparison to non-cardiac MIS-C patients. However, other biochemical parameters such as CRP, hemoglobin, sodium and ferritin levels were not significantly different between the cardiac and non-cardiac children.

### 3.3. MIS-C Children with Cardiovascular Abnormalities Have Elevated Cytokines and Chemokines 

**Figure 2:**
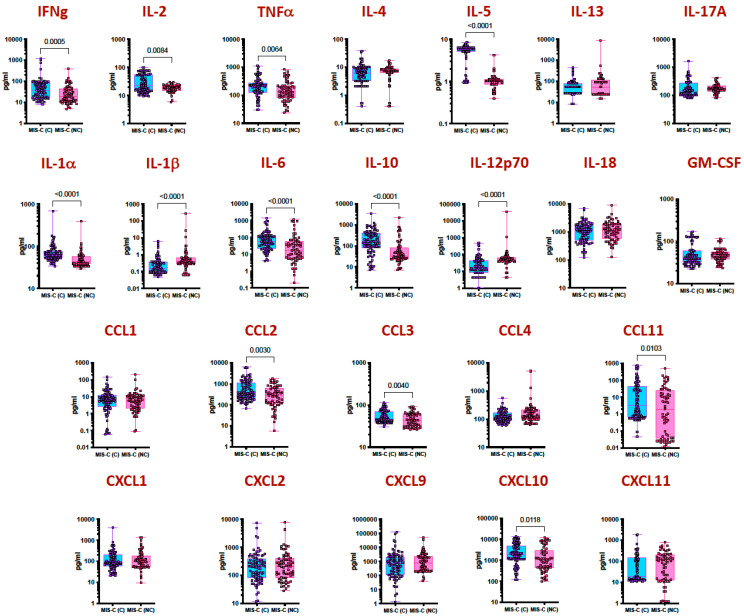
MIS-C children with cardiac involvement have elevated cytokines and chemokines. Plasma levels from MIS-C cardiac (MIS-C (C) (*n* = 88)) and MIS-C non-cardiac (MIS-C (NC) (*n* = 64)) children. Type 1, type 2, type 17, other proinflammatory cytokines, CC chemokines and CXC chemokines were measured using multiplex assays. Each circle represents a single individual, and the bars represent the geometric mean values. *p* values were calculated using the Mann–Whitney test with Holm’s correction for multiple comparisons.

To elucidate the differences in the cytokine and chemokine responses between MIS-C cardiac and MIS-C non-cardiac children, we assessed their levels in plasma. As shown in Figure 2, MIS-C cardiac children exhibited significantly increased production of type 1 (IFN-γ, IL-2, and TNFα), type 2 (IL-5) and other pro-inflammatory cytokines (IL-1α, IL-1β, IL-6, IL-10 and IL-12p70) in comparison to MIS-C non-cardiac children. In addition, as shown in Figure 1, MIS-C cardiac children exhibited significantly increased production of CC chemokines (CCL2, CCL3, and CCL11) and CXC chemokines (CXCL10) in comparison to MIS-C non-cardiac children.

### 3.4. Signature Pattern of Elevated Plasma Cytokines and Chemokines in MIS-C Children with Cardiovascular Abnormalities

**Figure 3:**
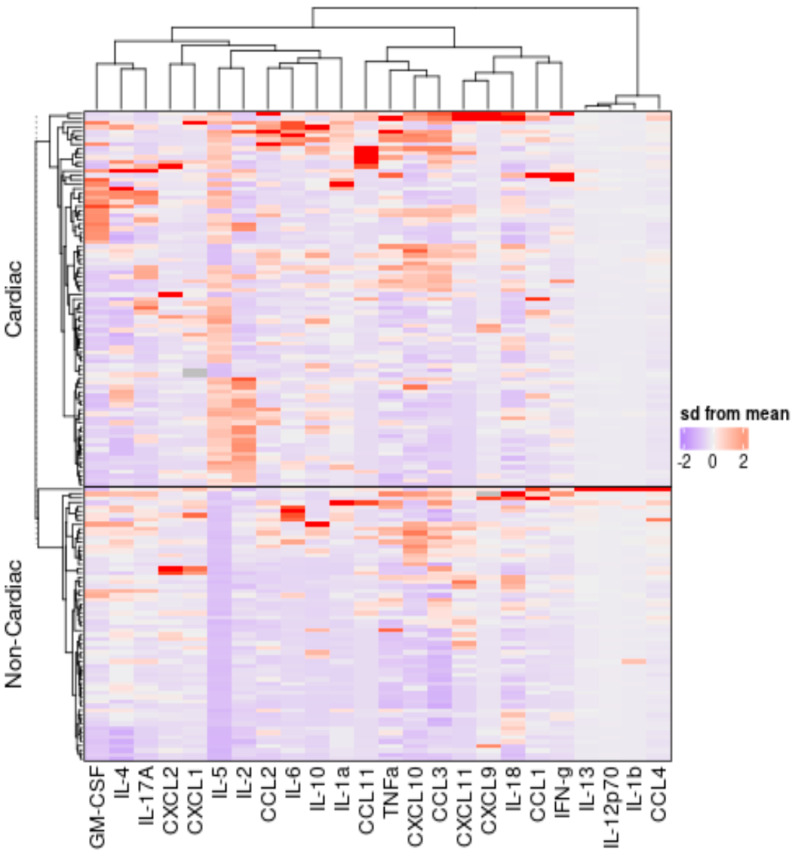
Cytokine and chemokine profiling of immune markers in children with MIS-C with and without cardiac manifestations. A hierarchical cluster analysis was performed to analyze the immune markers that were found to be statistically different exclusively in MIS-C children with and without cardiac manifestations and the univariate analysis could separate the groups according to individual levels of each subject. Data were log10 transformed and z-score normalized.

To measure the relationship and identify a signature pattern in our cohort of MIS-C children with cardiovascular manifestations, we estimated a panel of cytokines and chemokines in both groups of MIS-C children. Main analyses showed that several immune parameters presented statistically different concentrations in plasma between the study groups. As shown in Figure 3, data were z-score normalized across the entire cohort and analyzed using hierarchical clustering methods like “EUCLIDEAN distance” and “WARD.D2 method”. The cytokine names are shown on the left side of the heatmap. The “Complex Heatmap” package in “R Studio” software was used to visualize and observe the difference in the expression patterns of each of the cytokines and chemokines across the groups. The clusters were then identified, and these clusters showed clear distinction for MIS-C cardiac vs. MIS-C non-cardiac, albeit with some overlap. The cluster with cardiac involvement was prominent for higher expression of most of the analytes tested in the study. In the cardiac group compared to the non-cardiac group, the levels of cytokines (IL-1α, IL-1β, IL-6, IL-10 and IL-12p70) and chemokines (CCL2, CCL3, CCL11, and CXCL10) were significantly elevated. We therefore determine that, in our cohort, a signature pattern of the systemic level of cytokines and chemokines distinguished MIS-C cardiac from MIS-C non-cardiac children.

### 3.5. Associations between Cytokine/Chemokines and Other Biochemical Parameters

Next, we wanted to identify correlations between cytokine/chemokines and other biochemical parameters in both groups. We applied Spearman’s correlation coefficients to verify the correlation effect, and data were visualized according to heat map color intensity with variables being ordered through hierarchical clustering. A multiparametric matrix correlation plot exhibited good statistically significant correlations between circulating levels of cytokines/chemokines and biochemical parameters. As shown in Supplementary Materials Figure S1A, in the MIS-C cardiac group, cytokines (IL-1α, IL-2, IL-5, IFNγ, IL-1β, IL-12, IL-13, and IL-17A) and chemokines (CCL4, CCL11, and CXCL1) correlated well with biochemical parameters, namely total Pro BNP, troponin, WBC count, hemoglobin, ferritin, CRP and potassium. Meanwhile, in the MIS-C non-cardiac group, only a minimal correlation was seen (Supplementary Materials Figure S1B) (the cytokines IL-1α and IL-6 and the chemokines CXCL1 and CXCL2 with the biochemical parameters total WBC count and creatinine). Similarly, as shown in Supplementary Materials Figure S1C, in the combination of cardiac and non-cardiac group, cytokines such as GM-CSF, IL-1α, IL-2, IFNγ, IL-4 IL-6, IL-10, IL-12, and IL-17A and chemokines such as CCL1 and CCL11 were correlated with biochemical parameters such as total WBC count, hemoglobin, ferritin, CRP, sodium, potassium and creatinine.

### 3.6. Plasma Cytokine and Chemokine Levels Are Significantly Diminished Following MIS-C Treatment

**Figure 4:**
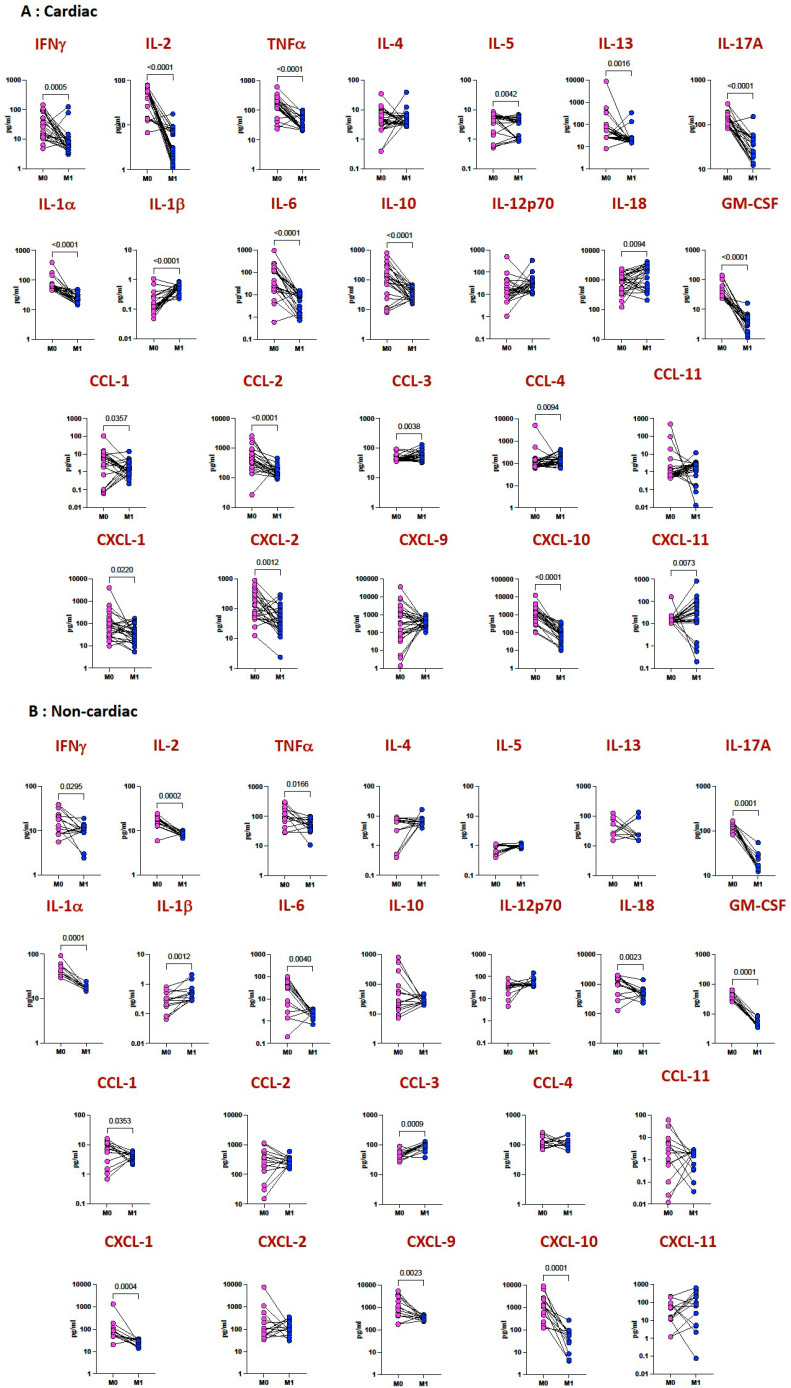
Diminished cytokine and chemokine levels after treatment and recovery. The plasma levels of cytokines and chemokines were measured in (**A**) MIS-C children with cardiac manifestations (*n* = 26) and (**B**) without cardiac manifestations (*n* = 14) at baseline (pre) and at 9 months following treatment (post). The data are presented as line graphs with each line representing a single individual. *p* values were calculated using the Wilcoxon signed rank test.

To examine whether the elevated plasma levels of cytokines and chemokines are directly linked with MIS-C, we measured the level of these cytokines and chemokines in both groups before and after treatment (pre versus post) in a subset of children. As shown in Figure 4A, at 9 months following treatment and recovery, the cytokine levels of INFγ, IL-2, TNFα, IL-13, IL-17A, IL-1α, IL-6, IL-10, IL-18 and GM-CSF and the chemokine levels of CCL1, CCL2, CCL3, CCL4, CXCL1, CXCL2, CXCL10 and CXCL11 were significantly diminished in MIS-C children with cardiac features compared to pre-treatment levels. Similarly, as shown in Figure 4B, at 9 months following treatment and recovery, the cytokine levels of INFγ, IL-2, TNFα, IL-17A, IL-1α, IL-6, IL-18 and GM-CSF and the chemokine levels of CCL1, CCL3, CXCL1, CXCL9 and CXCL10 were significantly diminished in MIS-C children with non-cardiac features compared to pre-treatment levels.

## 4. Discussion

We describe the immune profile of MIS-C children with or without cardiovascular involvement and identify immune biomarkers/signatures that could help to differentiate cardiac and non-cardiac presentation of MIS-C. It is well known that elevated levels of cytokines have systemic effects and in turn cause organ dysfunction [14], and this has been established in MIS-C [9]. However, the precise immune dysfunction still remains vague. As MIS-C mostly arises after a delay period following SARS-CoV-2 infection, it is assumed to be caused by unusual cellular or humoral adaptive immune responses eliciting inflammation or mediating organ damage [15,16]. The most frequent cardiac symptoms of MIS-C are left ventricular dysfunction, followed by electric conduction abnormalities and coronary artery aneurysm [17]. Severe myocardial dysfunction has been reported as a common cardiac abnormality in children with MIS-C [18]. Studies have also reported that coronary abnormalities were seen in 45.9% of MIS-C children, and the majority of cases display left ventricular dysfunction normalized before discharge [19]. A recent study from Poland reported that the majority of children had echocardiographic features of cardiovascular involvement in the course of MIS-C, but the majority of children with acute heart failure improved significantly within a few days of treatment [20]. The precise mechanism which leads to myocardial dysfunction in MIS-C is not yet fully understood. Possible reasons appear to be multifactorial, and may be secondary to cytokine storm, including generalized systemic hyper inflammation, vasculitis and possibly acute stress [18]. Cytokine release syndrome, also called “cytokine storm”, is illustrated by significantly higher inflammatory and proinflammatory cytokines such as IL-6 [8]. This phenomenon of increased inflammatory markers and systemic hyperinflammation is constantly found in children with MIS-C [7,9,14,21]. Through our study, we report the MIS-C children with cardiac manifestations exhibit even more elevated expression of proinflammatory cytokines such as IFNγ, IL-2, TNFα, IL-1α, IL-6 and IL-10 and anti-inflammatory cytokines such as IL-5 in comparison to MIS-C children without cardiac features. This increased cytokine surge possibly suggests cardiac injury through several hypothesized mechanisms including cardiomyocyte injury as a result of a severe and dysregulated inflammatory response [22]. In addition, our correlation analysis also clearly revealed that MIS-C children with cardiac manifestations showed a good correlation between the cytokines/chemokines and biochemical parameters, especially Pro BNP and troponin. This finding is also supported by published reports from Europe, which also revealed that children with cardiac involvement showed elevated levels of Pro BNP, ferritin, D-dimers, and cardiac troponin [8,23]. In addition, more specifically, CRP, an important marker of inflammation, is well correlated with cytokines/chemokines, indicating that MIS-C children with cardiac involvement exhibit a hyperinflammatory state.

Our previously published study [9] reported that MIS-C among Indian children is characterized by elevated levels of cytokines (IFNγ, IL-2, TNFα, IL-1α, IFNα, IFNβ, IL-6, IL-15, IL-17A, GM-CSF, IL-10, and IL-33) and chemokines (CCL2, CCL19, CCL20 and CXCL10) at the systemic level as well as upon SARS-CoV-2 antigen stimulation [9,24], along with growth factors such as VEGF, acute phase proteins (CRP, α-2-Macroglobulin and serum amyloid P) and microbial translocation markers (LPS, sCD14, and LBP) [9,24] in the plasma of MIS-C children. In addition, our published findings also revealed that other inflammatory markers such as matrix metalloproteinases (MMPs), which are involved in lung pathology, were also significantly elevated in MIS-C [25] compared to COVID-19 or controls. MIS-C children with shock were characterized by higher levels of INFγ, TNFα, IL-6, IL-10, CRP, serum amyloid P and sCD14, confirming the distinct immunopathogenesis by correlating with the disease severity of MIS-C [9,26]. MIS-C may be related to a hyperimmune response to the virus in a genetically vulnerable younger population as the underlying cause of these elevated immune markers [2,3]. COVID-19 may cause cardiac injury through a variety of proposed pathways, including the viral invasion of cardiomyocytes that results in cellular damage, microvascular dysfunction, and cardiomyocyte injury caused by an acute and dysregulated inflammatory response related to cytokine storm [22]. Further to our previous reports, we now reveal from the clustering analysis that there is a clear discrimination of cytokines and chemokines between the MIS-C children with cardiovascular abnormalities in comparison to MIS-C children without cardiovascular abnormalities. This immunological evaluation is important as this could help to unravel disease severity and establish the clinical course in MIS-C children and may help clinicians in management.

In the case of similar chronic diseases like Kawasaki disease (KD), both KD and MIS-C are associated with a significant cytokine storm that results in systemic inflammation and may explain the myocardial dysfunction that is often seen in these patients. The elevated levels of ferritin and troponins are some of the surrogate markers for MIS-C [27]. Another overlapping clinical presentation similar to MIS-C is children with acute febrile illnesses like dengue and systemic lupus erythematosus (SLE). The presence of mucocutaneous features and highly elevated CRP could distinguish MIS-C from dengue [28], while SLE has rarely been found in children during the COVID-19 pandemic [29]. The current understanding of MIS-C is evolving, with the majority of publications detailing immunological results prior to therapy commencement. Studies on the post-treatment immunological responses are limited [10]. The follow up of MIS-C patients, especially children, with cardiovascular manifestations is important in determining possible sequelae. Immunological findings using sequential measurements of both cell-mediated and cytokine immune responses may offer insight into disease pathogenesis and treatment [15]. Previous studies have reported that immunologic disturbances noted during the acute phase of illness normalize over time [11,30]. Our present findings add to the existing literature by showing that circulating cytokines and chemokines levels fall with time. Our findings suggest that the exaggerated pro-inflammatory responses in MIS-C are normalized following treatment and recovery and immune homeostasis is achieved in these children. However, the long-term significance of this finding is yet to be established. In our cohort, children were treated with intravenous immunoglobulins (IVIG), corticosteroids, and rarely IL-6 inhibitor (Tocilizumab). IVIGs can neutralize the immunological effect of autoantibodies, and the use of steroids may provide broad immunosuppression. However, it is not yet clear whether both are required in non-cardiac MIS-C. Similarly, there is no consensus on the choice of biological agents (IL-1 antagonists vs. IL-6 receptor blockers vs. anti-TNF agents). In our opinion, combined IVIG and steroids may be beneficial in MIS-C cardiac children, as they together will reduce tissue inflammation or prevent the progression of coronary artery aneurysm/myocardial dysfunction faster. However, the elevated immune responses in MIS-C cardiac children compared to MIS-C non cardiac children has been demonstrated, implying that MIS-C cardiac children may require greater immunosuppression. Studies with larger sample size are required to validate our hypotheses. Our study suffers from the limitation of examining children from a single city and only MIS-C children without non-MIS-C controls. In addition, there is also a lack of comparison with Kawasaki syndrome. Our study was also descriptive in nature and did not delineate underlying mechanisms, implying that further research is warranted to provide definitive answers.

Our data here propose a novel direction for imminent work towards the mechanistic understanding of the immunopathology in MIS-C and its underlying immune perturbation. We believe that the strengths of this study are the use of well-characterized participant groups (MIS-C cardiac vs. non-cardiac) with a good number of study participants. Our current basic scientific investigations into MIS-C and the host immune response have provided additional clues related to understanding the pathogenesis of this syndrome. Finally, to our knowledge, this is the first study to show that plasma cytokines and chemokines can be used as tools in determining disease severity in MIS-C children with cardiac manifestations in India. The detailed analysis in our study provides additional information and may guide clinicians in the assessment and management of children presenting with cardiac abnormalities in MIS-C children. In conclusion, our research adds to the amount of information already available and increases our understanding of the immunopathogenesis of MIS-C in children.

## Figures and Tables

**Table 1:** Study demographics and clinical characteristics.

	Cardiac *n* = 88	Non-Cardiac *n* = 64	*p* Value
Age median (years, IQR)	6 (2–9 y)	6.2 (3–10 y)	*p* = 0.5544
Male *n* (%)	55 (62%)	33 (51%)	0.178
Underlying conditions *n* (%)	2 (5%) ^£^	4 (3%) ^a^	-
Symptoms *n* (%)			
Fever	88 (100%)	64 (100%)	-
Breathlessness	62	6	*p* < 0.0001
Cardiovascular symptoms/signs Shock Hypotension Coronary artery dilatation Mycocardial dysfunction	70 (80%) 74 (84%) 22 (25%) 44 (50%)	5 (8%) 3 (5%) 0 0	*p* < 0.0001 *p* < 0.0001
Laboratory parameters			
CRP (<3 mg/L)	24 (12–48.0)	24 (12–91.5)	*p* = 0.29
Hemoglobin (g/dl) Median (IQR)	10.2 (9.2–10.8)	10.5 (9.1–11.6)	*p* = 0.15
Lymphocyte (/mm^3^) (1500–4000) median (IQR)	9200 (5800–11000)	2280 (1027–3944)	*p* < 0.0001
Platelets (200–450) × 10^9^/L median (IQR)	180 (121–435)	145 (53–352)	*p* < 0.0001
Sodium (135–145 mmol/L) median (IQR)	131 (128–134)	132 (124–135)	*p* = 0.42
Ferritin (ng/mL) (7 to 140) median (IQR)	611 (263–1345)	427 (195–775)	*p* = 0.6
ProBnP (pg/mL) (<250) median (IQR)	5553 (570–22000)	667 (421–3350)	*p* < 0.05
Troponin (ng/L) (<19) median (IQR)	28.6 (2–150)	1.5 (0.01–106)	*p* < 0.05
Treatment *n* (%)			
IVIG	47 (53%)	19 (30%)	0.00358
Steroids	73 (83%)	52 (81%)	0.786
PICU admission	86 (98%)	20 (31%)	<0.0001
Tocilizumab (8 mg/kg)	1	1	-
Mortality *n* (%)	2	1	-

^£^ Lymphoma, seizure disorder, ^a^ acute lymphoblastic leukemia, seizure disorder, juvenile idiopathic arthritis, diabetes.

## Data Availability

All the reported data are available within the manuscript.

## Supplementary Materials

The following supporting information can be downloaded at: https://www.mdpi.com/article/10.3390/v15112162/s1. Figure S1. Relationship between Immune markers and biochemical parameters.
