# Supplementary material for: Immune Profiles in Multisystem Inflammatory Syndrome in Children with Cardiovascular Abnormalities
Source: Viruses. 2023 Oct 27;15(11):2162. doi: 10.3390/v15112162 (PMC10674423; doi:10.3390/v15112162)
Supplement: Supplementary file 1 [file viruses-15-02162-s001.zip › viruses-2578196-supplementary.pdf]

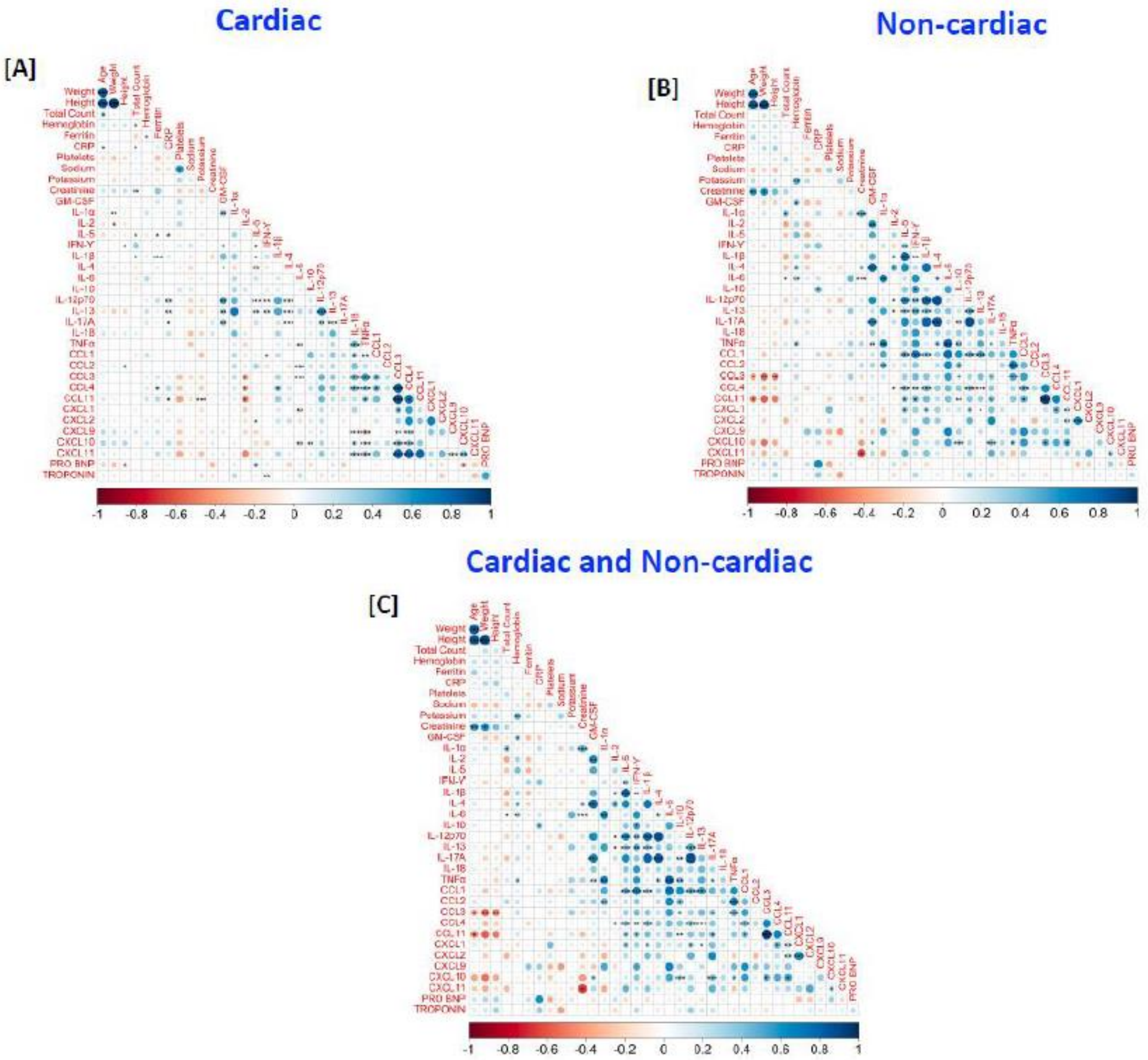

Figure S1. Relationship between Immune markers and biochemical parameters. Multiparametric matrix correlation plot of immune markers and biochemical parameters in all individuals of MIS-C with and without cardiac manifestations. Spearman's correlation coefficients are visualized by colour intensity. P values are ordered by hierarchical clustering.
